# Clinical Features, Outcomes, and Risk Factors of Bloodstream Infections due to *Stenotrophomonas maltophilia* in a Tertiary-Care Hospital of China: A Retrospective Analysis

**DOI:** 10.1155/2019/4931501

**Published:** 2019-12-09

**Authors:** Yibing Chen, Jijiang Suo, Mingmei Du, Liangan Chen, Yunxi Liu, Leili Wang, Zhixin Liang

**Affiliations:** ^1^Department of Respiratory Medicine, Chinese PLA General Hospital, Beijing 100853, China; ^2^Department of Infection Management and Disease Control, Chinese PLA General Hospital, Beijing 100853, China; ^3^Department of Microbiology, Chinese PLA General Hospital, Beijing 100853, China

## Abstract

*Background. Stenotrophomonas maltophilia* bacteremia (SMB) is the most perilous situation as compared to other types of *S. maltophilia* infection. The present study aimed to investigate the clinical features, distribution, drug resistance, and predictors of survival of SMB in a tertiary-care hospital of China. *Methods*. SMB that occurred in a tertiary-care hospital in Beijing, China, within 9 years (2010–2018) was investigated in a retrospective study. Demographics, incidence, commodities, drug resistance, mortality, as well as antibiotics administration were summarized according to the electronic medical records. The risk factors for survival were analyzed by Chi-square test, Kaplan–Meier curve and Cox regression. *Results*. A total of 76 episodes of SMB were analyzed. The overall incidence of SMB fluctuated from 3.4 to 15.4 episodes per 1000 admissions over 9 years. Malignancy was the most common comorbidity. High *in vitro* sensitivity was observed to minocycline (96.1%), levofloxacin (81.6%), and trimethoprim-sulfamethoxazole (89.5%). Central venous catheter (CVC) (*p* = 0.004), mechanical ventilation (MV) (*p* = 0.006), hemodialysis (*p* = 0.024), and septic shock (*p* = 0.016) were significantly different between survival and death group. The 30-day mortality was 34.2% within 30 days after confirmation of blood culture. Factors such as hemodialysis (OR 0.287, 95% CI: 0.084–0.977, *p* = 0.046), T-tube (OR 0.160, 95% CI: 0.029–0.881, *p* = 0.035), and septic shock (OR 0.234, 95% CI: 0.076–0.719, *p* = 0.011) were associated with survival. *Conclusions*. *S. maltophilia* is the major nosocomial blood stream infectious pathogenic bacteria. Trimethoprim-sulfamethoxazole and minocycline are optimal antibiotics for the treatment of SMB. T-tube, hemodialysis, and septic shock were the risk factors associated with survival of SMB patients.

## 1. Introduction

*Stenotrophomonas maltophilia *(*S. maltophilia*) is a Gram-negative, glucose non fermentative bacterium, widely existing in nature and hospital environment. Recently, it has emerged as a significant opportunistic pathogen of nosocomial infections, according to the data of WHO [1]. Due to its weak invasiveness, *S. maltophilia* commonly causes infections related to immunological deficiency in critically ill patients, especially lower respiratory tract infection, but also for urinary system, blood stream, abdominal, and skin and soft tissue infections, resulting in up to 37.5% mortality [2]. The intrinsic resistance of the bacteremia to specific antibiotics renders the clinical treatment difficult. Therefore, in the cases of *S. maltophilia* bacteremia (SMB), the 30-day mortality was 53.3% [3, 4]. The literature reported certain risk factors for *S. maltophilia* infection in Korea and Canada [4]. Nevertheless, SMB in China manifests a different spectrum antibiotic susceptibility in vulnerable population [2, 10]. Thus, it is imperative to have a detailed insight into the characteristics, risk factors as well as drug resistance of SMB in China. Therefore, we analyzed the clinical data of patients with SMB within 9 years (2010–2018) in a tertiary care, teaching hospital, focused on risk factors associated with the survival rate and drug resistance, thereby providing evidence on the predictors of mortality and appropriate treatment of SMB patients in China.

## 2. Methods

### 2.1. Study Population and Design

This retrospective study was conducted in a 2200-bed tertiary care hospital in Beijing, China, from January 2010 to December 2018. This hospital had six intensive care units (Intensive care unit, ICU) containing more than 100 beds. All hospitalized patients were tested positive for SMB in venous blood cultures from a total of 76 patients. Infections monitored by the Department of Infection Management and Disease Control and identified through the microbiological laboratory during the hospital stay were qualified for this study. The following characteristics and clinical data of patients were recorded from electrical medical records: demographics, underlying diseases, risk factors for SMB, Acute Physiology and Chronic Health Evaluation II (APACHE II) score for ICU patients, occurrence of severe sepsis or septic shock, infection distribution, time from admission to onset, overall time for hospitalization, antibiotics susceptibility testing results, antibiotic therapy, and overall mortality (within 30 days).

Blood was cultured using a BacT/AlerT 3D automatic blood detection system (Becton–Dickinson, Sparks, MD, USA), and positive cultures were inoculated with an automated agar plate inoculation system (PREVI Isola; bioMérieux, Marcy l'Etoile, France). The presence of *S. maltophilia* was confirmed using the VITEK-2 system (bioMérieux). Antibiotic susceptibility testing was performed using the VITEK 2 system or the Kirby–Bauer disk diffusion method (Oxoid, UK) according to the recommendations proposed by the Clinical and Laboratory Standards Institute (CLSI) [5, 27], and the results were obtained from the first positive blood culture specimens.

### 2.2. Definition

SMB was defined as one or more episodes of positive blood culture in a patient combined with clinical symptoms of systemic inflammatory response syndromes [6]. An episode was defined as at least one blood culture sample from a patient without prior blood culture isolating the same bacteria within the previous 30 days. Onset of SMB was defined as the date when the blood culture was collected. In the case of patients with multiple positive blood cultures, the first result was included in the data collection. Polymicrobial infections were defined as two or more other bacterial infections except for *S. maltophilia*. Nosocomial infection was defined as SMB occurring ≥48 h after admission [7]. Prior antibiotic use was defined as any antibiotic treatment for >24 h within 1 month before the episode of infection [8] Systemic corticosteroid therapy was defined as 1 week of treatment with prednisone minimum at 1 mg/kg/day or equivalent before the diagnosis of SMB. The central venous catheter (CVC) removal was defined as within 5 days after blood drawing for culture. Appropriate definitive antibacterial therapy was defined as the targeted regimen that included at least one antibiotic agent to which *S. maltophilia* was susceptible *in vitro*. This antibiotic therapy began within 5 days after positive blood culture of *S. maltophilia*. A combination therapy was defined as any two of the three antibiotics: trimethoprim/sulfamethoxazole (TMP/SMZ), Minocycline and Levofloxacin. Multi-drug resistance was defined as *in vitro* resistance to three or more types of antibiotics.

### 2.3. Statistical Analysis

Data were analyzed using SPSS 22.0 statistics (IBM, Armonk, NY, USA). Demographic and clinical data were routinely described and analyzed. Continuous variables were described as mean (standard division, SD), categorical variables were described as frequency counts or percentages (*n*, %). Median and Student's *t*-test were used for continuous variables, while the Chi-square test or Fisher's exact test was used for categorical variables. The risk factors were initially estimated using Chi-square test and the Kaplan–Meier method for univariate analysis, in order to screen possible factors related to mortality or survival. The variables (*p* < 0.10) were statistically related to the 30-day mortality in the univariate analyses used to construct the multivariate model. Cox regression analysis was performed to assess the factors associated with the 30-day mortality. A two-sided *p*-value < 0.05 was considered to be statistically significant.

## 3. Results

### 3.1. Demographic and Clinical Characteristics of the Patients

A total of 76 blood samples obtained from 76 patients from 1 Jan, 2010 to 31 Dec, 2018 were positive for *S. maltophilia* culture. The age of the patients ranged from 4–87 (average, 54.1) years, and 26 patients (34.2%) were >65-year-old. The cohort consisted of 69.7% (53/76) males. The average of the length of hospital stay was 67.64 (range, 1–500) days, and the mean duration of hospitalization before identification of *S. maltophilia* infection was 41.75 (range, 3–233) days. In addition, 92.1% (70/76) patients showed associated febrile response, with temperature >38℃ during hospitalization. 25% patients were admitted to the ICU ward; all the patients were given antibiotics before developing an *S. maltophilia* bacteremia, either for a previous other infection or as empirical treatment for sepsis (Table 1).

The comorbidities were identified as listed in Table 1, the top three include: pulmonary infection (25/76, 32.9%), cardiovascular diseases (22/76, 28.9%) and diabetes mellitus (15/76, 19.7%). A total of 73.7% (56/76) patients had polymicrobial infections. The most common coexisting microorganisms were *Acinetobacter baumanii* (53.5%, 30/56), *Candida albicans* (48.2%, 27/56), *Pseudomonas aeruginosa* (35.7%, 20/56), *Klebsiella pneumoniae* (32.1%, 18/56), and *Escherichia coli* (30.4%, 17/56).

### 3.2. Incidence and Sources of Infection

**Figure 1:**
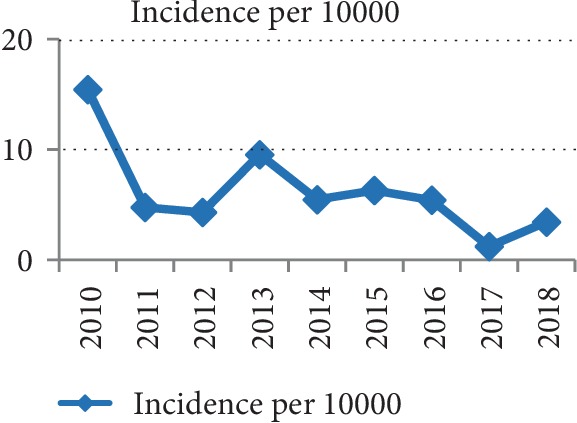
Incidence of blood stream infection due to *S. maltophilia* from 2010 to 2018.

The total number of 76 specimens was obtained from 76 patients. The overall incidence of SMB was 5.7 episodes/10,000 admissions, and the rate fluctuated from 1.2 to 15.4 episodes/10,000 admissions during the 9 years (15.4 in 2010, 4.8 in 2011, 4.3 in 2012, 9.5 in 2013, 5.4 in 2014, 6.3 in 2015, 5.4 in 2016, 1.2 in 2017, and 3.4 in 2018) (Figure 1). Except for one patient, the rest were all nosocomial infections. The infection onset time ranged from 3 to 233 (average, 41.75) days after hospital admission. Also, we counted 49 positive *S. maltophilia* cultures for non-bloodstream infection in the 76 patients: catheter tip (6/49, 4 were from great vein catheter and 2 from central venous catheter), sputum (24/49), pharynx swab (1/49), nose swab (1/49), ear swab (1/49), wound swab (2/49), cerebrospinal fluid (2/49), ascites (5/49), bile (5/49), and urine (2/49). Thus, we can conclude that the respiratory infection maybe the major source of blood stream infection.

### 3.3. Distribution of Infections

Of the total 76 patients, 16 were found in surgery wards, including the Department of Hepatobiliary Surgery and Neurosurgery; 41 were found in internal medicine wards, including the Department of Respirology, Cardiology, Hematology, Gastroenterology, Neurology, and Pediatrics; 19 were found in ICU.

### 3.4. In Vitro Susceptibility

Susceptibility testing was performed for three known susceptible antibiotics during 5 years, according to the international guidelines, as exhibited in Table 2. High *in vitro* sensitivity was observed to minocycline (96.1%), levofloxacin (81.6%) and trimethoprim-sulfamethoxazole (89.5%). Only six isolates were resistant to levofloxacin, 3 to TMP/SMZ, 3 to both levofloxacin and TMP/SMZ, 3 showed intermediate resistance to minocycline, 5 to levofloxacin, and 1 to TMP/SMZ. Only 36.8% (28/76) patients were treated with susceptible antibiotics, including trimethoprim-sulfamethoxazole (TMP/SMZ) (4/28, 14.3%), minocycline (1/28, 3.6%), and levofloxacin (15/28, 53.6%). A total of 7 patients adopted a combined treatment, including TMP/SMZ+minocycline (2/28), minocycline+levofloxacin (4/28), and TMP/SMZ+levofloxacin (1/28). In addition, we detected the susceptibility to the other three common antibiotics; cefoperazone (23/57, 40.4%), ceftazidime (35/67, 52.2%), and ciprofloxacin (42/76, 55.3%).

### 3.5. Risk Factors for Mortality

The risk factors for SMB are listed in Table 3. The overall mortality was 34.2% (26/76), and 38.5% (10/26) was associated with septic shock. Nearly 1/2 of the mortality occurred within 30 days after the positive culture results. Among these risk factors, mechanical ventilation (MV), hemodialysis, septic shock, and CVC differed significantly between the survival and death groups, which is in accordance with some previous studies.

**Figure 2:**
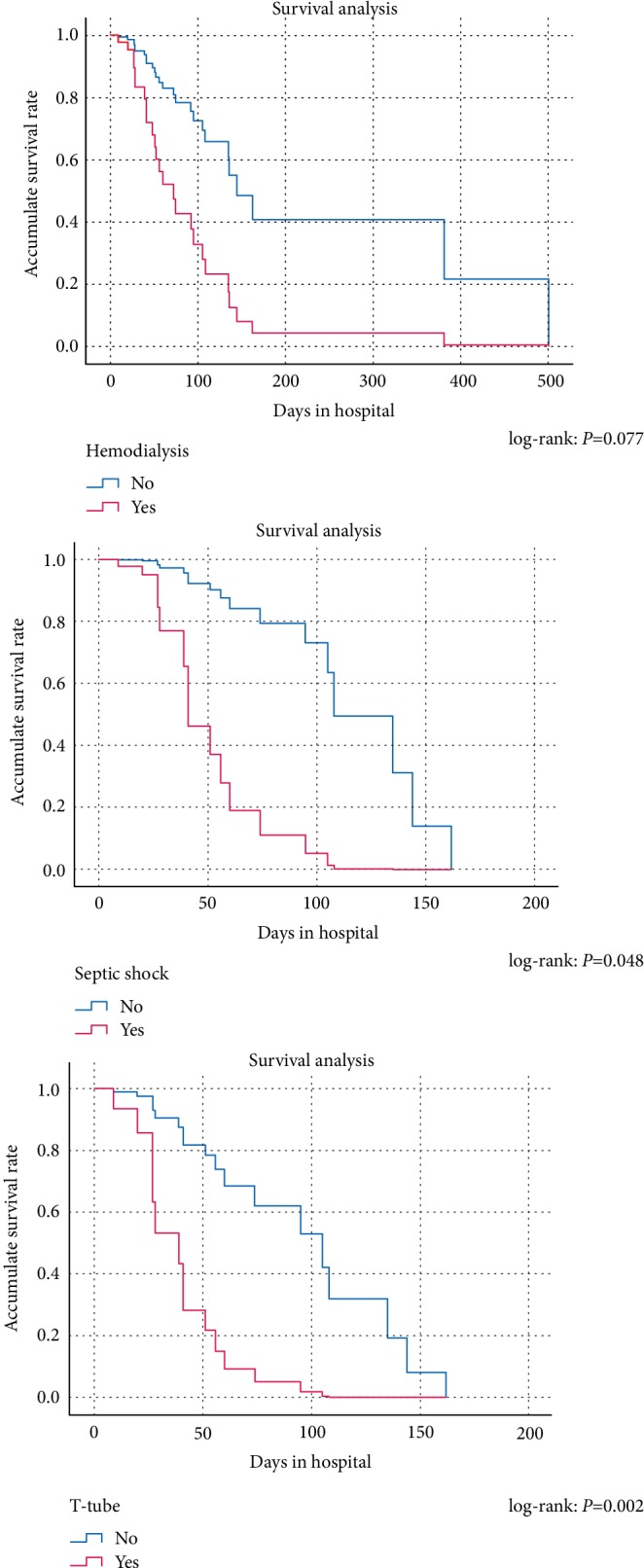
Kaplan–Meier curve comparing the survival rate between patients with and without hemodialysis, septic shockand T-tube.

According to the Kaplan–Meier survival analysis for univariate analysis (Figure 2), CVC, T-tube, hemodialysis, septic shock and total parenteral nutrition were significantly associated to the survival rate. However, further multivariate Cox regression analysis showed that T-tube, hemodialysis, and septic shock were risk factors associated with the survival rate of SMB patients (Table 4). Active antibiotic treatment after SMB confirmation did not show any effect on the ultimate mortality.

## 4. Discussion

Since the last decade, *S. maltophilia* has emerged as the third most common non fermentative Gram-negative bacillus responsible for nosocomial infections, preceded by *P. aeruginosa* and *Acinetobacter* spp. Previously, the bacterium isolated in the lower respiratory tract has been regarded as colonization rather than as infection [9, 10, 11]. In this retrospective study, 76 SMB cases were reviewed from 2010 to 2018 in a large comprehensive teaching hospital in China. To the best of our knowledge, this is the first SMB analysis mainly in Chinese, and we concluded that: (i) the characteristics of patients with SMB were similar to other studies abroad; (ii) MV, hemodialysis, septic shock, and CVC were associated with patients' survival; (iii) hemodialysis, T-tube, and septic shock were risk factors for the survival of SMB patients.

In the current study, the majority of patients, except for trauma patients, had more than one underlying diseases. The all-cause mortality within 30 days was 34.2%, similar to some uncontrolled clinical trials [2, 12, 13]. Patients with SMB exhibit some common clinical characteristics as described previously [14], such as long hospital stay, long-time venous passage presence, immunosuppressive condition, and high comorbidity of malignancy. Although we have 19 patients from ICU, the majority were from public wards, encompassing internal medicine to surgical medicine wards. Furthermore, the main factors of significant difference between death and survival group were CVC, mechanical ventilation, hemodialysis, and septic shock, similar to that descried previously [15]. Irrespective of the underlying diseases, these four risk factors affected the all-cause mortality. The Cox regression analysis showed that hemodialysis, T-tube and septic shock were risk factors associated with the survival time, and the initial disease condition of the patients contributed more than SMB on the all-cause mortality [12].

Noticeably, the incidence of SMB showed a declining trend from 2010 to 2018. This might be explained with the increased importance of prevention and control infection among medical staff over recent years. Besides, the sample size was small, further verification with a larger sample size is required. Furthermore, in terms of the source of infection, the traceable sources of SMB primarily originated from sputum (49.0%, 24/49), i.e., the respiratory pathway. Thus, we should pay attention to the disinfection of the air in our hospital, especially in the critically ill patients' wards.

In the current study, the most frequently occurring primary diseases were malignancies, including those in the hematological system (14/76) and other solid tumors (18/76), followed by the digestive system (13/76) and trauma (8/76). Moreover, the most common comorbidities were pulmonary infection (25/76, 32.9%), cardiovascular diseases (22/76, 28.9%), and diabetes mellitus (15/76, 19.7%). The primary characteristics of the patients included the presence of CVC (76.3%), previous administration of corticosteroids (50.0%), urethral catheter (44.7%), and mechanical ventilation (35.5%). In some studies, SMB is considered to be related to the presence of CVC [8]; CVC removal could reduce mortality [15]. However, the current study showed that CVC removal did not affect the prognosis of SMB patients (*p* = 0.102). T-tube drainage was often used in patients with bile duct obstruction. In addition, we found 10.2% (5/49) *S. maltophilia* infection in bile source, and T-tube is also one of the risk factors associated with the survival rate. Since bile was a common source of *S. maltophilia*, the presence of T-tube might imply poor prognosis in patients with bile duct infection.

Almost all the patients (98.7%) had received antibiotic treatment before the onset of *S. maltophilia* infection. While after the confirmation of SMB, only 36.8% (28/76) patients received susceptible antibiotics (Table 5), which was a rather low ratio. One of the reasons we found was that a large number of patients were discharged before the blood culture results were obtained. For the treatment of SMB, levofloxacin was the most common used antibiotic (78.6%, 22/28), while trimethoprim-sulfamethoxazole and minocycline were also used in 25% patients, respectively. However, we found that the mortality rates were almost same between patients who did and did not receive appropriate therapy. Whether susceptive antibiotics therapy can increase the survival rate post-*S. maltophilia* infection remains controversial. In a previous review study, the inappropriate antimicrobial therapy did not exert a significant impact on mortality [12]. Another northwest Chinese study [11] also showed the same result, although the main type of infection was lower respiratory tract infection. Surprisingly, a Japanese study [16] demonstrated that patients without specific treatment for *S. maltophilia *showed a paradoxically higher survival rate than those who received treatment.

Reportedly, the prior use of carbapenem is a risk factor of *S. maltophilia* infection [17, 18, 19] due to its intrinsic resistance to the drug. In this study, 58 (76.3%) patients were given carbapenem prior to SMB diagnosis, which might serve as the guidance to select the antibiotics, especially for the treatment of patients with severe illness. Approximately, 80% (58/76) of the patients in our study were administered carbapenem before the diagnosis of SMB infection, and 90% were given more than one antibiotic for the treatment of infections. Therefore, clinicians should note that *S. maltophilia* infection might occur after the administration of broad-spectrum antibiotics, especially in severely ill patients with co-infection of other bacterial species.

Drug-resistant test showed that the majority of *S. maltophilia* infection was susceptible to TMP/SMZ (89.5%), minocycline (96.1%), and levofloxacin (81.6%) (Table 2). The levofloxacin resistance rate (10/76, 13.2%) was relatively low as compared to Korea, but much higher than the USA [13]. Drug resistance was often detected for TMP/SMZ or levofloxacin, and no resistance to minocycline was found in our study. In a Korean study [23], prior levofloxacin exposure for 3 weeks was independently associated with levofloxacin drug-resistance in *S. maltophilia*. Moreover, due to the similar 30-day mortality, some investigators suggested that levofloxacin can be an useful alternative option for treating SMB [24]. A Taiwanese study [22] concluded that previous piperacillin/tazobactam treatment was administered often in the levofloxacin-resistant group. Thus, levofloxacin is a valuable choice for SMB treatment. TMP/SMZ is recommended as first-line therapy in *S. maltophilia* infection. Resistance to TMP/SMZ was >5% as reported previously from the Asia-Pacific region (8% of strains resistant) and Europe (10%) [20]. The current study showed that TMP/SMZ is preferable in China.

Notably, we have two pediatric infections in this study; one was diagnosed with neurogenic tumor, and another with hematological malignancy. *S. maltophilia* has been reported as an emerging pathogen in the pediatric population [25, 26]. Although similar report was rare in China, this special population should be under intensive focus.

## 5. Conclusion

A total of 76 Chinese cases of SMB from 2010 to 2018 were summarized in a retrospective study. Consequently, SMB that was associated with poor prognosis, was mostly a nosocomial infection. The most effective antibiotics for SMB were trimethoprim-sulfamethoxazole and minocycline. Patients who underwent hemodialysis, T-tube drainage, and experienced septic shock had a significantly shorter survival than those who did not undergo the same.

## Figures and Tables

**Table 1:** Overall demographic and clinical characteristics of S. maltophilia Infected patients.

Characteristics	*n* (%)
Age (years); median ± SD (range)	54.1 ± 21.1 (4–87)
Male gender	53 (69.1)
Comorbidities	
Cardiovascular disease	22 (28.9)
Solid tumor	14 (18.4)
Diabetes mellitus	15 (19.7)
Hematologic malignancy	14 (18.4)
Pulmonary infection	25 (32.9)
Nervous system diseases	14 (18.4)
Cholangitis	12 (15.8)
Severe acute pancreatitis	6 (7.9)
Sever trauma	5 (6.6)
Chronic renal failure	3 (3.9)
Aplastic anemia	3 (3.9)
Femoral fracture	2 (2.6)
Prior hospital stay, median ± SD (range)	41.7 ± 49.3 (3–233)
Overall hospital stay, median ± SD (range)	67.6 ± 85.3 (1–500)
ICU residence	19 (25.0)
Distribution of infections	
Surgery wards	16 (21.1)
ICU	19 (25.0)
Internal medicine wards	41 (53.9)

**Table 2:** Susceptibility pattern of 76 tested *S. maltophilia* isolates.

Antimicrobial agents	Susceptible (%)	Intermediate (%)	Resistant (%)
TMP/SMZ (*n* = 76)	68 (89.5)	1 (1.3)	7 (9.2)
Minocycline (*n* = 76)	73 (96.1)	3 (4.0)	0
Levofloxacin (*n* = 76)	62 (81.6)	4 (5.2)	10 (13.2)
Cefoperazone (*n* = 57)	23 (40.4)	10 (17.5)	24 (42.1)
Ceftazidime (*n* = 67)	35 (52.2)	5 (7.5)	27 (40.3)
Ciprofloxacin (*n* = 76)	42 (55.3)	7 (9.2)	17 (22.4)

**Table 3:** Risk factors related to 30-day mortality of SMB^∗^.

Variables	Total (*n* = 76)	Survived (*n* = 50)	Death (*n* = 26)	*p*
*Age (years)*				
≥65	26	15	11	
<65	50	35	15	0.966^a^
*Gender*				0.653^a^
Male	53	34	19	
Female	23	16	7	
*Risk factors for SMB * ^c^:				
CVC∗	58	33	25	0.004^b∗^
Thoracic tract	8	4	4	0.434^b^
Abdominal tract	20	12	8	0.525^b^
T-tube	9	6	3	0.100^b^
Urinary tract	34	19	15	0.145^b^
Trachea intubation	21	12	9	0.419^b^
Tracheotomy	8	3	5	0.114^b^
MV∗	27	13	14	0.006^b^ ∗
Surgery (within 30 days)	28	15	12	0.209^b^
Chemotherapy	23	19	4	0.566^b^
Hemodialysis	19	8	11	0.024^b∗^
Septic shock	10	3	7	0.016^b∗^
Previous corticosteroids	38	23	15	0.469^b^
Total parenteral nutrition	12	5	7	0.055^b^
ICU residence	19	10	9	0.163^b^
APACHE Ⅱ score, median		14.1 ± 8.3	22.4 ± 7.7	
Mean ± SD	11	10	1	0.065^a^
Charlson comorbidity index	76	3.5 ± 3.7	4.2 ± 3.3	0.315^a^
Prior use of antibiotics∗ mean ± SD	76	5.1 ± 3.1	6.6 ± 3.4	0.068^a^
Co-infection		29	19	0.196^b^

^a^: *t*-test; ^b^: Pearson's chi-squared test; ^c^: SMB = *Stenotrophomonas maltophilia* bacteremia; CVC = central venous catheter; MV = mechanical ventilation; Prior use of antibiotics = types of antibiotics used prior to SMB was confirmed.

**Table 4:** Multivariate Cox regression model analysis.

Factors	30-day mortality		95.0% CI for Exp (B)	
	*p*	OR	Lower	Upper
CVC	0.106	0.182	0.023	1.438
MV	0.161	2.405	0.705	8.201
Hemodialysis	0.046	0.287	0.084	0.977
T-tube	0.035	0.160	0.029	0.881
Septic shock	0.011	0.234	0.076	0.719
Susceptible Antibiotic use	0.290	1.642	0.656	4.113

OR: odds ratio; CI: confidence interval; ICU: intensive care unit.

**Table 5:** Antibiotic use after SMB confirmation.

Antibiotics	*n* (%)
TMP/SMZ	4 (5.3)
Minocycline	2 (2.6)
Levofloxacin	15 (19.7)
Combination therapy	7 (9.2)
Other antibiotics	10 (14.7)

## Data Availability

The datasets generated during the current study are not publicly available to avoid disclosure of the individual privacy of the patients, but are available from the corresponding author on reasonable request. Jijiang Suo is an equal contributor.
